# The Dutch Individualised Care Scale for patients and nurses – a psychometric validation study

**DOI:** 10.1111/scs.12853

**Published:** 2020-04-16

**Authors:** Sofie Theys, Ann Van Hecke, Reinier Akkermans, Maud Heinen

**Affiliations:** ^1^ Faculty of Medicine and Health Sciences Department of Public Health and Primary Care – University Centre for Nursing and Midwifery Ghent University Ghent Belgium; ^2^ IQ Healthcare Radboud University Medical Centre Nijmegen The Netherlands; ^3^ Nursing Science and Allied Healthcare Radboud Institute for Health Sciences IQ Healthcare Radboud University Medical Centre Nijmegen The Netherlands

**Keywords:** Individualised Care Scale, individualised care, psychometrics, reliability, validity, nursing

## Abstract

**Aims and objectives:**

Translating and psychometrically assessing the Individualised Care Scale (ICS) for patients and nurses for the Flemish and Dutch healthcare context.

**Background:**

Individualised care interventions have positive effects on health outcomes. However, there are no valid and reliable instruments for evaluating individualised care for the Flemish and Dutch healthcare context.

**Design:**

Psychometric validation study.

**Setting and participants:**

In Flemish hospitals, data were collected between February and June 2016, and in Dutch hospitals, data were collected between December 2014 and May 2015. Nurses with direct patient contact and a working experience of minimum 6 months on the wards could participate. Patient inclusion criteria were being an adult, being mentally competent, having an expected hospital stay of minimum 1 day, and being able to speak and read the Dutch language. In total, 845 patients and 569 nurses were included.

**Methods:**

The ICS was translated into Dutch using a forward–backward translation process. Minimal linguistic adaptations to the Dutch ICS were made to use the scale as a Flemish equivalent. Omega, Cronbach’s Alpha, mean inter‐item correlations and standardised subscale correlations established the reliability and confirmatory factor analysis the construct validity of the ICS.

**Results:**

Internal consistency using Omega (Cronbach’s Alpha) ranged from 0.83 to 0.96 (0.82–0.95) for the ICS‐Nurse and from 0.88 to 0.96 (0.87–0.96) for the ICS‐Patient. Fit indices of the confirmatory factor analysis indicated a good model fit, except for the root mean square error of approximation, which indicated only moderate model fit.

**Conclusion:**

The Dutch version of the ICS showed acceptable psychometric performance, supporting its use for the Dutch and Flemish healthcare context.

**Relevance to clinical practice:**

Knowledge of nurses’ and patients’ perceptions on individualised care will aid to target areas in the Dutch and Flemish healthcare context in which work needs to be undertaken to provide individualised nursing care.

## Introduction

Since the professional development of the nursing practice by Florence Nightingale, one of the premises of nursing care has been the patient’s individuality (1). Respect for the individuality and uniqueness of all persons receiving nursing care is considered mandatory according to the International Council for Nurses. Due to the shift from the biomedical model to the holistic paradigm over the last century, there has been an increasing attention towards tailored healthcare interventions and individualised care in clinical practice and research (2). Literature indicates that individualised nursing care is considered important by both nurses and patients (3), and has the potential to improve healthcare quality. A number of studies have shown that individualised nursing has a positive effect on patient satisfaction with nursing care (4, 5, 6, 7), mobility, recovery, and self‐care ability (8), and patients’ reported quality of life (7, 9). It also has the capacity to decrease healthcare‐associated costs (10). Further, a systematic review on job satisfaction for professionals showed some positive effects on general job satisfaction, job demands, emotional exhaustion and personal accomplishment among professionals delivering individualised care (11).

Suhonen et al. (12) have developed and psychometrically evaluated the Individualised Care Scale (ICS), which permits measuring the perception on individualised care of nurses and hospitalised patients. In this study, a Dutch translation and psychometric evaluation of the ICS for patients and nurses were carried out to establish whether the Finnish model also fits the data retrieved from patients and nurses in the Flemish and Dutch hospital settings.

## Background

The concept of individualised care is one of the many variations in the terminology used to define patient‐centred care (13, 14), and various tool for measuring the concept of patient‐centred care exist (14, 15, 16). Measurement tools attempt to measure either the holistic concept or specific subcomponents such as shared decision‐making (13). The rapid review of de Silva (14) indicates that the commonly used measurement tools in published research about the broad holistic concept of patient‐centred care are as follows: the Measure of Processes of Care, the Person‐centred Care Assessment Tool, the Person‐centred Climate Questionnaire and the ICS. Yet, they are of no better quality than other measurement instruments, as studies that compare the merits of different measures are lacking (14). In the systematic review of Köberich et al. (15), four instruments that measure perceptions of patient‐centred nursing care were reported: the ICS, the Client Centred Care Questionnaire, the Oncology Patients’ Perceptions of the Quality of Nursing Care Scale and the Smoliner Scale. This study will focus on the validation of the ICS developed by Suhonen et al. (12, 17). Of the above‐mentioned measurement tools, the ICS is the most generic measurement instrument that focuses on the broad holistic concept of patient‐centred care. Furthermore, the ICS allow to measure both nurses’ and hospitalised patients’ perceptions on individualised care.

Suhonen et al. (18) define individualised nursing care as nursing care that takes into account the individuality of the patient and facilitates patient participation in decision‐making. Research suggests that patients vary substantially in their preferences for participation in decision‐making, ranging from preferring to co‐decide, to fully relying on the clinical expertise of their health provider (19). Providing individualised care means assessing differences in patient characteristics, preferences and perceptions, and tailor healthcare interventions accordingly (19, 20, 21).

### The Individualised Care Scale

The ICS is a Finnish, bi‐partite, Likert‐type scale that allows the assessment of both nurses’ and hospitalised patients’ perceptions on individualised nursing care by means of two separate ICS scales, namely the ICS‐Patient and the ICS‐Nurse (12, 17). Each scale contains 34 items, divided into two subsections. For the ICS‐Patient, the first section (ICSA‐Patient) consists of 17 items and was designed to measure patients’ views on how individuality was supported through specific nursing interventions. The second section (ICSB‐Patient) consists of 17 items and measures how patients perceive individuality in their care. Both sections consist of three subscales that consecutively measure (i) patient characteristics in the clinical situation (ClinA and B, seven items), (ii) the patient’s personal life situation (PersA and B, four items) and (iii) decisional control over care by the patient (DecA and B, six items). The scale is formatted into a five‐point Likert scale (1 = fully disagree, 2 = disagree, 3 = neither disagree nor agree, 4 = agree, 5 = fully agree). The ICS was mirrored in order to measure nurses’ perceptions on (i) how they support patients’ individuality through specific nursing activities (ICSA‐Nurse) and (ii) the evaluation of maintaining individuality in their provided care (ICSB‐Nurse). Both sections also consist of three subscales: (i) clinical situation (ClinA and B, seven items); (ii) personal life situation (PersA and B, four items); and (iii) decisional control over care (DecA and B, six items). A higher score on the ICSA section indicated that nursing activities were perceived as highly individualised. A higher score on the ICSB section indicated a higher perception of individuality in patients’ care (12, 22).

Individualised care is considered to be one of the key characteristics in assessing quality of care. A proper translation and validation of the ICS is necessary in order to determine whether the scale can be used in its original form or needs adaptations due to cultural differences. This also could enhance the assessment of cross‐cultural effects of individualised healthcare interventions on clinical outcomes. Currently, the ICS has been translated in English, Greek, German, Turkish, Swedish, Spanish and Portuguese and used in several international studies (5, 23, 24, 25, 26, 27, 28). There have been no previous studies that measured patients’ and nurses’ perceptions on individualised care conducted in Flanders and the Netherlands. Measuring both patients’ and nurses’ perceptions on individualised care will aid to identify the extent to which nurses and patients share the same understanding of the care provided (29, 30). This study focused on translating the ICS for Flanders and the Netherlands and assessed its reliability and construct validity through confirmatory factor analysis on Dutch data from both nurses and patients.

## Methods

### Translation of the Individualised Care Scale

The ICS was translated from English into Dutch, using the forward–backward translation procedure. The English ICS was translated into Dutch independently by two senior researchers with adequate skills in English (C1 level) and with profound expertise in individualised health care. One independent certified English linguist translated the Dutch ICS back to English. The original ICS and the back‐translated ICS were compared, and semantic alterations to the Dutch scale were made accordingly. For the ICS‐Nurse, alterations were made to seven items and for the ICS‐Patient to ten items (e.g. from ‘The nurses talked with me about the feelings I have had about my condition’ to ‘The nurses talked with me about my feelings regarding my condition’). Minimal linguistic adaptations to the Dutch ICS were made to use the scale in Belgium as a Flemish equivalent. For both the ICS‐Nurse and the ICS‐Patient, alterations were made to seven items. Adaption from Dutch to Flemish was carried out by a group of two Flemish senior and two junior researchers in nursing science. Item content validity (I‐CVI) was established by asking five patients to judge the wording and comprehensibility of the items, and seven students following a master’s programme in nursing sciences (combining the programme with a job in nursing care) reviewed the items regarding comprehensibility, relevancy and linguistic correctness using the content validity indexing technique. It was opted to use master’s students because they were able to assess the comprehensibility and relevancy of the items from their position as a student researcher and their position as a nurse. First, the I‐CVI was calculated by dividing the number of raters giving a rating of either 3 or 4 on the 4‐point Likert scale (ranging from totally disagree to totally agree), by the total number of raters (31). However, as the I‐CVI does not, on its own, correct for chance agreement among the raters, a formula that integrates an I‐CVI score into a modified kappa statistic calculation that corrects for chance was used (32). The modified kappa evaluation criteria are as follows: Fair 0.40–0.59; Good 0.60–0.73; and Excellent_0.74 (32). Of the items, 9% were rated as fair, 18% as good and 73% as excellent. In this study, both versions, the Dutch (The Netherlands) and the Flemish (Belgium) ICS scale, were considered as one single scale, because of its minor differences. We therefore refer to the scale as the Dutch ICS.

### Psychometric evaluation of the Individualised Care Scale

#### Setting

For the validation of the Dutch ICS, data collected in Flemish (Flanders) and Dutch hospitals (The Netherlands), participating in two improvement projects to enhance patient participation in hospitals (the implementation of bedside shift reporting and the implementation of the Tell‐us card) were used. Flemish hospitals are situated within the Dutch‐speaking, Flemish Community (Flanders) of Belgium. No hospitals of the French‐speaking, Walloon Community (Wallonia) of Belgium were included.

In Flanders, quality coordinators, chief nursing officers and chief medical officers from all Flemish regional hospitals (n = 68) and university hospitals (n = 8) were invited to engage in the improvement projects. Exploratory meetings took place with head nurses, chief nursing officers, and chief medical officers to discuss eligibility in the study. Wards for surgery, geriatric care, internal medicine, medical rehabilitation and maternal care were included. Hospitals willing to participate had to give an informed consent signed by the chief executive officer. [Correction added on 20 Sep 2020, after first online publication: both n values have been corrected in this paragraph]

In the Netherlands, three surgical wards and one cardiology ward residing within the same university hospital and one cardiology ward of a regional hospital were invited to engage in the study. Exploratory meetings took place with ward managers to discuss eligibility in the study. Hospitals willing to participate had to give an informed consent signed by the ward manager.

In total, nurses on 34 wards and patients on 29 wards of two hospitals in the Netherlands and ten hospitals in Flanders participated in the improvement projects. An overview of all included wards per hospital and per improvement project is presented in Table 1.

**Table 1:** Overview of all included wards per hospital and per study

Study	Hospital	Discipline	Nurses per hospital (n)	Patients per hospital (n)
Tell‐us Card Flanders	Hospital A	General surgery 1, General surgery 2, General surgery 3	35	101
	Hospital B	General surgery, Oncology, Maternity	53	101
	Hospital C	Maternity	16	30
	Hospital D	Locomotor rehabilitation, Heart and Lung Diseases, Neurology/Nephrology	33	56
	Hospital E	Maternity	20	39
	Hospital F	Neural rehabilitation	11	6
	Hospital G	Maternity	17	34
Tell‐us Card the Netherlands	Hospital H	Neurosurgery, Head and Neck surgery, Orthopaedics, Cardiology	63	109
	Hospital I	Cardiology	42	24
Bedside Shift Report Flanders	Hospital A	Locomotor rehabilitation, Neural rehabilitation	25	39
	Hospital B	Orthopaedics/General surgery/Rheumatology, Orthopaedics/Traumatology, Locomotor rehabilitation, Neurosurgery	70	115
	Hospital C	Geriatrics	20	N/A
	Hospital G	Geriatrics, General surgery	46	41 (Geriatrics N/A)
	Hospital J	Geriatrics	35	N/A
	Hospital K	Cardiopulmonary rehabilitation, Orthopaedics, Neurology	40	63
	Hospital L	Cardiopulmonary rehabilitation, Neural rehabilitation/Physiology, Orthopaedics	43	87
	Total		569	845

N/A, Not available.

#### Participants

Nurses with direct patient contact and a working experience of at least 6 months on the ward were eligible for participation in the studies. Adult patients (age > 18) mentally competent with adequate ability to speak and read the Dutch language and with an expected hospital stay of at least 1 day were included. Being mentally competent was assessed by the nurses of the ward. Patients who had trouble remembering, learning new things, concentrating and making decisions due to medication side effects, delirium, depression, dementia and other mental illnesses were excluded. Also, patients who were intellectually disabled due to trauma before birth, trauma during birth, inherited disorders and chromosome abnormalities were excluded.

#### Data collection

In Flanders, data were collected between February and June 2016, and in the Netherlands, data were collected in between December 2014 and May 2015. A list of the hospitalised patients who met the inclusion criteria was available for the researchers. In Flanders, the ICS for the patients was distributed by a member of the research team and recollected after 2 hours. If patients did not have the opportunity to complete the questionnaire in time, a collection box was available on the ward. If patients were in the impossibility of filling in the questionnaire themselves due to motoric difficulties, a member of the research team or sometimes a study nurse with no affiliation to the research team assisted the patient by filling in his answer. In the Netherlands, patients received a questionnaire with a prepaid return envelope to be filled in at home after discharge.

The questionnaire for the nurses was distributed in a closed envelope. By weekly visits to the wards (in the Netherlands by regular visits and weekly emails), nurses were reminded of filling in the questionnaire. A collection box was available on the ward. After 1 month, the questionnaires were collected by a member of the research team. Nurses who did not fill in the questionnaire upon collection were addressed personally by the head nurse and again invited to participate.

### Analysis

Statistical analyses were performed using SPSS 25 (SPSS Inc, Chicago, IL, USA), R statistical software packages and AMOS 22 (SPSS Inc). Descriptive statistics (percentages, means and SDs) were reported to describe both patients’ and nurses’ socio‐demographic characteristics. To check whether the missing items were missing (completely) at random, it was compared whether the socio‐demographic characteristics of the respondents (nurses/patients) with missing data differed from those of the respondents (nurses/patients) without missing data. The full sample of nurses and patients was recoded into a group of respondents with at least one missing item on the ICS scale and a group of respondents without missing items. Characteristics of the group of respondents with at least one missing item and the group of respondents with no missing items were compared using chi‐squared tests and *t*‐tests.

The reliability of the subsections and the subscales was examined in relation to the instrument’s internal consistency by calculating both Omega and Cronbach’s Alpha coefficients, and the homogeneity of the instrument (mean inter‐item correlations, item‐to‐total correlations and standardised subscale correlations). As Cronbach’s alpha has been shown to be unrelated to a scale’s internal consistency and a fatally flawed estimate of its reliability, it is more appropriate to report Omega (33). However, other studies assessing the internal consistency reliability of the ICS always report the Cronbach’s alpha. Therefore, Cronbach’s alpha was also reported in this study. This allows to compare the internal consistency reliability of the Dutch ICS with those reported in other studies.

The matrix of adequate internal consistency in light of item count and sample size provided by Ponterotto and Ruckdeschel (34) was used to determine the relative strength of the Omega and Cronbach’s alpha coefficients. Mean inter‐item correlations situated within a 0.30–0.70 range were considered satisfactory (35). Item‐to‐total correlations were acceptable against the criteria of r > 0.30 (36).

Construct validity was investigated using structural equation modelling in the form of a confirmatory factor analysis. An a priori assumption of the underlying structure Suhonen et al. (17), with two subsections that each contains three corresponding subscales, was made. Factor loadings and standard errors were reported. Factor loadings that exceeded the criterion of 0.30 were regarded as good indicators of the respective subscales (37). Because the chi‐square statistics may be inflated by larger sample sizes and is no longer relied upon as a basis for acceptance or rejection, fit indices which are less dependent on sample size were interpreted (38, 39). A comparative fit index (CFI) >0.90 suggests a good model fit, while values >0.95 suggest an excellent model fit. For the standardised root mean square residual (SRMR), values lower than 0.08 indicate a good model fit. For the root mean square error of approximation (RMSEA), values of less than 0.07 indicate good model fit (40, 41, 42). Model modifications on the basis of modification indices or the Lagrange multiplier test were conducted (43). Modification indices showed that model fit would improve if certain items were allowed to correlate (43). Consistent with the recommendations of Hermida (44), the number of possible error correlations was limited to a minimum, allowing only error correlations between items that were similar in formulation or meaning.

### Ethical considerations

This study was approved by the ethical committee of the participating hospitals (Blinded) and (blinded). Informed consent was obtained from all patients and nurses through provision of detailed information on the purpose of the improvement project (Tell‐us card or Bedside shift reporting) and the confidentiality.

## Results

### Patients’ and nurses’ characteristics

Due to no differences in characteristics between patients (nurses) with at least one missing item and patients (nurses) without missing items on the ICS, data from patients (nurses) with one or more missing values (for patients 193 cases and for nurses 37 cases) were eliminated. In total, 845 patients from eleven hospitals and 569 nurses from twelve hospitals were included in the analysis. The sample size is sufficiently large to give adequate power for the statistical analyses, as the recommendation is using a sample that is ten to twenty times the number of parameters to be estimated in the confirmatory analyses (31, 40).

The mean age of the patients was 57 (SD = 19.3). More than half of the patients were females (57%). Most patients (71.1%) lived together with a partner, friend or family, had an education lower than bachelor’s degree (66%) and were retired (46%). The average amount of days of hospital admission was 11.2 days (SD = 17.4).

Nurses were on average 40 (SD = 12.5) years old and mostly female (90%). Half of the nurses had a bachelor degree (51%), 42% had a vocational degree and almost 3% had a university degree. About 4% of the participants were nursing assistants. Most nurses had 1–5 years of work experience (24%) or 20 or more years of work experience (31%) and were fully employed (43%). An overview of all patients’ and nurses’ characteristics is presented in Table 2.

**Table 2:** Descriptive statistics for patients and nurses

	Patients (n = 845)	Nurses (n = 569)
Age Mean (SD)	56.5 (19.3)	39.7 (12.5)
Days of hospital admission Mean (SD)	11.2 (17.4)	
Gender n (%)		
Male	363 (43.1)	58 (10.2)
Female	479 (56.9)	511 (89.8)
Level of education (patients) n (%)		
<Bachelor	551 (65.8)	
Bachelor	203 (24.3)	
Master	83 (9.9)	
Level of education (nurses) n (%)		
Nurse assistant^a^		22 (3.9)
Vocational nurse^b^		237 (42.1)
Bachelor educated^c^		288 (51.2)
Master educated^d^		16 (2.8)
Living condition n (%)		
Alone	185 (26.0)	
With a partner, family or friend	506 (71.1)	
In a service flat, assisted living or a nursing home	21 (2.9)	
Years of nurses’ working experience n (%)		
<1 year		28 (4.9)
1 to 5 years		139 (24.4)
6 to 10 years		107 (18.8)
11 to 15 years		71 (12.5)
16 to 20 years		48 (8.4)
>20 years		176 (30.9)
Work percentage		
<50%		87 (16.0)
50–99%		222 (40.9)
100%		234 (43.1)
Employment status		
Employed	277 (39.0)	
Unemployed	24 (3.4)	
Student	16 (2.3)	
Disabled	65 (9.1)	
Retirement	329 (46.3)	
Type of hospital		
University	325 (38.5)	186 (32.7)
Regional	520 (61.5)	383 (67.3)
Type of ward		
Internal medicine	178 (21.1)	125 (22.0)
Maternity	146 (17.3)	76 (13.4)
Geriatric	N/A	80 (14.1)
Surgical	300 (35.5)	160 (28.1)
Medical rehabilitation	192 (22.7)	103 (18.1)
Mixed surgical/internal	29 (3.4)	25 (4.4)

N/A, not available.

^a^ One year of education at level 3 of the European Qualifications Framework (EQF).

^b^ Three years of education at level 5 of the EQF to obtain a diploma in Nursing.

^c^ Three years of education at level 6 of the EQF to obtain the degree of Bachelor in Nursing.

^d^ Five years of education at level 7 of the EQF to obtain the degree of Master in Nursing

### Construct validity

To assess construct validity, a confirmatory factor analysis was carried out. All paths from the subscales to the items were statistically significant at the 5% level. For patients, standardised factor loadings ranged 0.61–0.85 (0.53–0.86) for ClinA‐Patient (ClinB‐Patient), 0.71–0.83 (0.58–0.83) for PersA‐Patient (PersB‐Patient) and 0.58–0.84 (0.50–0.86) for DecA‐Patient (DecB‐Patient). For nurses, factor loadings ranged 0.73–0.85 (0.64–0.87) for ClinA‐Nurse (ClinB‐Nurse), 0.63–0.76 (0.67–0.79) for PersA‐Nurse (PersB‐Nurse) and 0.62–0.83 (0.47–0.85) for DecA‐Nurse (DecB‐Nurse). An overview of the standardised factor loadings is provided in Tables 3 and 4. The CFI did reach the cut‐off value of >0.90 for the sample of patients on both subsections and for the sample of nurses on the ICSB subsection. The SRMR did reach the cut‐off value of <0.08 for both the sample of nurses and patients on both subsections. Contrastingly, the RMSEA did not reach the cut‐off value of <0.07 for both the sample of nurses and patients on both subsections. In Table 5, an overview of the fit indices is given.

**Table 3:** Standardised factor loadings for patients

Item content	Factor loadings ICSA			Factor loadings ICSB		
	1	2	3	1	2	3
1. Feelings about the condition	0.76			0.84		
2. Needs that require care and attention	0.76			0.81		
3. Opportunity to take responsibility in one’s own care as far as able	0.61			0.53		
4. Changes in the condition of the patient	0.69			0.81		
5. Fears and anxieties about the condition	0.83			0.85		
6. Effects the condition has on the patient	0.84			0.86		
7.Meaning of the illness for the patient	0.85			0.86		
8. Everyday life outside the hospital		0.76			0.83	
9. Previous experiences of hospitalisation		0.76			0.79	
10. Everyday habits		0.83			0.83	
11. Preferences for family involvement in care		0.71			0.58	
12. Receiving understandable instructions			0.70			0.50
13. Knowledge preferences about the condition			0.78			0.78
14. Patients’ wishes regarding their care			0.75			0.86
15. Opportunities for decision‐making in one’s own care			0.84			0.83
16. Opportunity for expressing opinions in one’s own care			0.84			0.84
17. Having a choice when to wash			0.58			0.53

ICSA, Individualised Care Scale – Scale A; ICSB, Individualised Care.

**Table 4:** Standardised factor loadings for nurses

Item content	Factor loadings ICSA			Factor loadings ICSB		
	1	2	3	1	2	3
1. Feelings about the health condition	0.81			0.84		
2. Needs that require care and attention	0.83			0.85		
3. Opportunity to take responsibility in one’s own care as far as able	0.73			0.64		
4. Identify changes in feelings regarding the condition	0.79			0.86		
5. Talk about fears and anxieties regarding the condition	0.85			0.86		
6. Find out how the condition affects the patient	0.78			0.87		
7. Find out what the illness means for the patient	0.78			0.84		
8. Everyday life outside the hospital		0.75			0.69	
9. Previous experiences of hospitalisation		0.67			0.68	
10. Everyday habits		0.76			0.79	
11. Family involvement in care		0.63			0.67	
12. Giving understandable instructions to the patient			0.73			0.69
13. Knowledge preferences of the patient about the condition			0.62			0.67
14. Patients’ wishes regarding the care			0.83			0.83
15. Helping patients take part in decisions concerning their care			0.81			0.79
16. Helping patients to express their opinions			0.75			0.85
17. Asking patients at what time they prefer to wash			0.67			0.47

ICSA, Individualised Care Scale – Scale A; ICSB, Individualised Care.

**Table 5:** Summary of model fit of the Dutch version of the ICS for nurses and patients

	Items (n)	Nurses			Items (n)	Patients		
		SRMR^c^	CFI^d^	RMSEA^e^		SRMR^c^	CFI^d^	RMSEA^e^
ICSA^a^	17	0.0524	0.893	0.103	17	0.0463	0.917	0.089
ICSB^a^	17	0.0391	0.945	0.076	17	0.0445	0.925	0.089
ICSA^b^	17	0.0484	0.922	0.089	17	0.0408	0.945	0.073
ICSB^b^	17	0.0377	0.959	0.066	17	0.0422	0.942	0.079

ICSA, Individualised Care Scale – Scale A; ICSB, Individualised Care.

^a^ Without *post hoc* modifications.

^b^ With *post hoc* modifications.

^c^ Standardised root mean square residual (an acceptable value is below 0.80).

^d^ Comparative fit index (an acceptable value is more than 0.90).

^e^ Root mean square error of approximation (an acceptable value is below 0.07)

Because (i) the correlation matrix of the reliability analysis showed high correlations (>0.70) between items 6 & 7 and 15 & 16, (ii) modification indices suggested adding error correlations between certain items and (iii) two experts in scale development agreed that items 6 & 7 and 15 & 16 had similar item content, error correlations were added between those items. This resulted in a better model fit. However, the RMSEA still did not reach the cut‐off value of <0.07 for the sample of nurses on the ICSA subsection and for the sample of patients on both subsections. An overview of the error correlations is given in Table 6.

**Table 6:** Overview of error correlations

	Items 6 & 7	Items 15 & 16
ICSA‐Patient	‘Made an effort to find out how the condition has affected me’ & ‘Talked with me about what the condition means to me’	‘Helped me take part in decisions concerning my care’ & ‘Helped me express my opinions on my care’
ICSB‐Patient	‘The way the condition has affected me has been taken into account in my care’ & ‘The meaning of the illness to me personally has been taken into account in my care’	‘I have taken apart in decision‐making concerning my care & ‘The opinions I have expressed have been taken into account in my care’
ICSA –Nurse	‘I make an effort to find out how their health condition has affected them’ & ‘I talk with patients about what the health condition means to them’	‘I help patients take part in decisions concerning their care’ & ‘I encourage patients to express their opinions on their care’
ICSB‐Nurse	‘I took into account the way the health condition has affected them’ & ‘I took into account the meaning of the health condition to the patient’	‘Patients took part in decision‐making concerning their care’ & ‘I took into account the opinions patients expressed about their care’

ICSA, Individualised Care Scale – Scale A; ICSB, Individualised Care Scale – Scale B.

### Internal consistency reliability

The Omega coefficients for ICS‐Nurse and the ICS‐Patient ranged from 0.83 to 0.96 and from 0.88 to 0.96. The Cronbach’s alpha coefficients for ICS‐Nurse and the ICS‐Patient ranged from 0.82 to 0.95 and from 0.87 to 0.96. Standardised subscale correlations ranged from 0.78 to 0.89 for the ICS‐Nurse and from 0.70 to 0.87 for the ICS‐Patient. All item‐to‐total correlations in both ICS‐Nurse and ICS‐Patient were higher than 0.30. Mean inter‐item correlations ranged from 0.50 to 0.68 for the ICS‐Nurse and from 0.52 to 0.63 for the ICS‐Patient. However, there was more variation in the individual inter‐item correlations. An overview of the values is displayed in Table 7.

**Table 7:** Summary of the reliability analysis of the Dutch version of the ICS for nurses and patients

	Nurses						Patients					
	Items (n)	Cronbach’s α	omega Ω	Mean inter‐item r	Item‐to‐total correlations	Subscale r	Items (n)	Cronbach’s α	omega Ω	Mean inter‐item r	Item‐to‐total correlations	Subscale r
ICSA	17	0.95 (0.95–0.96)	0.95 (0.95–0.96)	0.51 (0.29–0.77)	0.62–0.73		17	0.96 (0.95–0.96)	0.96 (0.95–0.96)	0.49 (0.28–0.82)	0.55–0.67	
ClinA	7	0.93 (0.92–0.94)	0.93 (0.93–0.94)	0.63 (0.49–0.77)	0.75–0.78	0.89	7	0.93 (0.93–0.94)	0.93 (0.93–0.94)	0.58 (0.44–0.76)	0.75–0.78	0.87
PersA	4	0.83 (0.81–0.85)	0.83 (0.81–0.85)	0.50 (0.42–0.58)	0.55–0.61	0.82	4	0.88 (0.86–0.89)	0.88 (0.86–0.89)	0.58 (0.49–0.65)	0.63–0.67	0.70
DecA	6	0.89 (0.87–0.90)	0.89 (0.88–0.90)	0.53 (0.29–0.71)	0.58–0.64	0.78	6	0.91 (0.90–0.92)	0.91 (0.90–0.92)	0.55 (0.39–0.82)	0.54–0.65	0.76
ICSB	17	0.95 (0.95–0.96)	0.96 (0.95–0.96)	0.52 (0.28–0.83)	0.44–0.77		17	0.96 (0.96–0.97)	0.96 (0.96–0.97)	0.52 (0.28–0.82)	0.50–0.79	
ClinB	7	0.95 (0.94–0.95)	0.95 (0.94–0.95)	0.68 (0.50–0.83)	0.81–0.83	0.86	7	0.95 (0.94–0.95)	0.95 (0.94–0.95)	0.63 (0.39–0.82)	0.80–0.81	0.86
PersB	4	0.82 (0.80–0.85)	0.83 (0.80–0.85)	0.50 (0.40–0.58)	0.54–0.60	0.78	4	0.87 (0.86–0.89)	0.88 (0.87–0.89)	0.56 (0.42–0.70)	0.51–0.76	0.85
DecB	6	0.89 (0.88–0.90)	0.89 (0.88–0.91)	0.54 (0.30–0.70)	0.44–0.68	0.86	6	0.91 (0.90–0.92)	0.91 (0.91–0.92)	0.52 (0.27–0.75)	0.47–0.51	0.78

r, Pearson’s correlation coefficient r, p < 0.001; Clin, clinical situation; Dec, decisional control over care; ICSA, Individualised Care Scale – Scale A; ICSB, Individualised Care Scale – Scale B; Pers, personal life situation.

## Discussion

Individualised care is an important aspect to be considered in providing qualitative nursing care and developing nursing care interventions (20). It is therefore essential to use a valid and reliable measuring instrument to assess both nurses’ and patients’ perceptions on how individualised care is provided. The Finnish Individualised Care Scale was developed and psychometrically validated to measure perceptions on individualised care in a Finnish healthcare context.

### Construct validity of the Dutch ICS

Confirmatory factor analysis supported evidence that the structure of the ICS corresponds to the Dutch sample data. The CFI did reach the cut‐off value of >0.90 for the sample of patients on both subsections and for the sample of nurses on the ICSB subsection. The SRMR did reach the cut‐off value of <0.08 for both the sample of nurses and patients on both subsections. However, even after allowing for error correlation between the items (i.e. item 6 & item 7; item 15 & item 16), the RMSEA did only reach the cut‐off value of <0.07 for the ICSB‐Nurse, indicating only moderate fit (45). The results of this study are similar to those of the German version of the ICS‐Patient, supporting evidence for the construct validity of the Dutch Individualised Care Scale. Values of the German version are (values for ICSB in parentheses) 0.090 (0.090) for the RMSEA, 0.092 (0.091) for the CFI and 0.05 (0.05) for the SRMR (26). However, fit indices of the Finnish ICS‐Nurse (values for ICS‐Patient in parentheses) showed a better model fit, with values of 0.062 (0.076) for the RMSEA, 1.00 (not reported) for the CFI and 0.015 (0.021) for the SRMR (17, 20).

All the factor loadings of the Dutch ICS exceeded the criterion of 0.30 and were therefore regarded as good indicators of their respective subscales (37). The factor loadings for the ICS‐Patient and the ICS‐Nurse subscales are similar to those in the studies of Suhonen et al. (17) and Suhonen et al. (12). When looking at the factor loadings in the cross‐cultural comparison study of Suhonen et al. (28), some factor loadings are lower in the Finnish, Greek, Swedish and British ICS compared with the factor loadings in the Dutch ICS.

### Internal consistency reliability of the Dutch ICS

Item‐to‐total correlations, inter‐item correlations and standardised subscale correlations supported evidence for the homogeneity of the ICS‐Nurse and ICS‐Patient for the Dutch sample data. All item‐to‐total correlations were acceptable against the criteria of r > 0.30. Mean inter‐item correlations were adequate against the criteria of 0.30–0.70, and the standardised correlations between subscales were all high indicating substantial similarity between subscales. Internal consistency using Omega and Cronbach’s alpha coefficients was good to excellent, with coefficients of 0.95–0.96 for the subsections and coefficients of 0.82–0.95 for the subscales (34). This indicates that the items of the tool fit together conceptually and represent the same phenomena within the sample. However, reliability coefficients over 0.9 might be an indication of redundancy in measuring intended construct within items (26, 46). Similar results were reported in other studies assessing the internal consistency of the ICS (23, 26, 27, 28). Suhonen et al. (28) suggested that given the high internal consistency for the subsections or subscales, it might be possible to shorten the questionnaire further. Based on the results of this study, high correlations (>0.70) between items 6 & 7 and items 15 & 16 and similar item content of these items, we suggest to shorten the questionnaire through deleting item 6 or 7 and item 15 or 16.

### Study limitations

Some limitations need to be taken into account in the interpretation of the results. First, data were collected on a range of various wards (surgical wards, internal wards, geriatric wards, maternity wards and rehabilitation wards). However, there was no sufficient power to do a hierarchical or stratified model with hospital ward as a variable (47). Second, in this study test–retest reliability as part of the evidence of ICS’s reliability was not assessed. This might be considered as a limitation of this study. Third, since only a small percentage of the items were rated as fair regarding content validity, it was decided to retain these items. However, this could have affected the construct validity of the Dutch version of the ICS and could be an explanation for RMSEA not reaching the cut‐off value of <0.07 for the sample of nurses on the ICSA subsection and for the sample of patients on both subsections (32). Fourth, adding post hoc inter‐item modifications might result in estimating data‐driven models that are potentially not generalisable across samples (48, 49). That is, the model may fit the particular data of the sample without a chance of being reproduced in other populations (50). Fifth, no patients were involved in judging the relevance of the ICS items. Nevertheless, the patient perspective had already been thoroughly examined in previous studies (20, 51). Last, no specific scales to assess cognitive impairment were used.

## Conclusion

Overall, the study on the Dutch version of the ICS showed adequate psychometric performance, supporting its use for the Dutch population. Internal consistency reliability was good, supporting the reliability of the scale. Moreover, acceptable model fit suggests that there is sufficient evidence to sustain the construct validity of the Dutch version of ICS.

## Relevance to clinical practice

Knowledge of nurses’ and patients’ perceptions on individualised care will help to target areas in the Dutch and Flemish healthcare context in which work needs to be undertaken to provide care adapted to the individuality of the patient and will help to be more aware of the obstacles to provide individualised nursing care (20, 52). Also, using a valid and reliable instrument to assess perceptions on individualised care for the Dutch and Flemish healthcare context will enhance clinical practice by allowing researchers and healthcare workers to develop individualised care interventions and measure their effects on several clinical and patient outcomes (20, 52).

## Conflicts of interest

No conflict of interest has been declared by the authors.

## Author contributions

All authors have contributed to the conception, design, analysis and interpretation of data for the work. All authors have contributed in drafting and revising the article for important intellectual content.

## Ethical approval

This study was approved by the Institutional Review Board of the study hospitals in Belgium (B670201526903) and the Netherlands (2014‐1350). Informed consent was obtained from all patients and nurses through provision of detailed information on the purpose of the improvement project (Tell‐us card or Bedside shift reporting) and the confidentiality.

## Funding

Funding for this research is granted by the Federal Public Service for Health, Food Chain Safety and Environment, and by the Ghent University. There was no involvement of this funding in this study. This research has received no further grants from any funding agency in the public, commercial or social profit sectors.
